# Aqua­(4,4′-bipyridine-κ*N*)bis­(1,4-dioxo-1,4-dihydronaphthalen-2-olato-κ^2^ *O* ^1^,*O* ^2^)zinc 4,4′-bipyridine mono­solvate dihydrate

**DOI:** 10.1107/S1600536811038682

**Published:** 2011-10-05

**Authors:** Marcos M. P. Silva, Lucas J. Carvalho, Maurício Lanznaster, Jackson A. L. C. Resende

**Affiliations:** a Departamento de Química Inorgânica, Instituto de Química, Universidade Federal Fluminense, Niterói, Rio de Janeiro, CEP 24.020-140, Brazil

## Abstract

The reaction of 2-hy­droxy-1,4-naphtho­quinone and 4,4′-bipyridine with zinc acetate produced the title compound, [Zn(C_10_H_5_O_3_)_2_(C_10_H_8_N_2_)(H_2_O)]·C_10_H_8_N_2_·2H_2_O. The bond lengths and angles around the metal atom indicate a deviation from octa­hedral geometry. The two naphtho­quinone ligands coordinate in a *cis* fashion, with the 4,4′-bipyridine ligand and the water mol­ecules completing the coordination sphere of the metal atom. The asymmetric unit contains also one free 4,4′-bipyridine mol­ecule and two uncoordinated water mol­ecules. These mol­ecules make contacts with the complex through O—H⋯N and O—H⋯O hydrogen bonds, creating a layer two-dimensional network parallel to (121).

## Related literature

For biological applications of naphtho­quinone-bearing complexes, see: Francisco *et al.* (2008 ▶); Bustamante *et al.* (2009 ▶); Neves *et al.* (2009 ▶). For reference structural data, see: Garge *et al.* (1990 ▶); Beni *et al.* (2006 ▶).
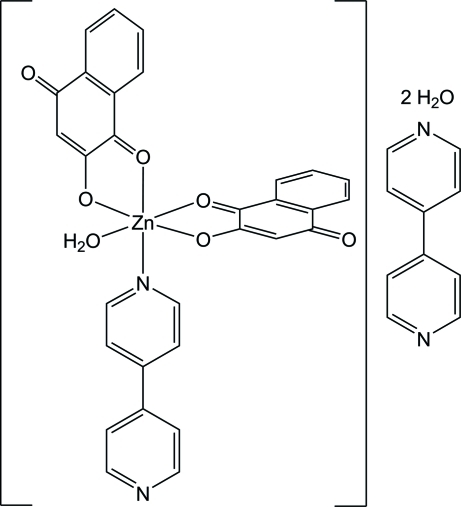


## Experimental

### 

#### Crystal data

[Zn(C_10_H_5_O_3_)_2_(C_10_H_8_N_2_)(H_2_O)]·C_10_H_8_N_2_·2H_2_O
*M* *_r_* = 778.07
Triclinic, 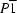

*a* = 8.1448 (16) Å
*b* = 14.179 (3) Å
*c* = 15.910 (3) Å
α = 73.90 (3)°
β = 88.59 (3)°
γ = 89.53 (3)°
*V* = 1764.8 (6) Å^3^
*Z* = 2
Mo *K*α radiation
μ = 0.76 mm^−1^
*T* = 293 K
0.18 × 0.12 × 0.10 mm

#### Data collection

Nonius Kappa CCD diffractometer
Absorption correction: multi-scan (*SORTAV*; Blessing, 1995 ▶) *T*_min_ = 0.983, *T*_max_ = 1.000
11283 measured reflections
6230 independent reflections
3619 reflections with *I* > 2σ(*I*)
*R*_int_ = 0.060

#### Refinement

*R*[*F* ^2^ > 2σ(*F* ^2^)] = 0.044
*wR*(*F* ^2^) = 0.089
*S* = 0.91
6230 reflections
487 parameters
H-atom parameters constrained
Δρ_max_ = 0.26 e Å^−3^
Δρ_min_ = −0.42 e Å^−3^

### 

Data collection: *COLLECT* (Nonius, 1998 ▶); cell refinement: *DIRAX/LSQ* (Duisenberg, 1992 ▶); data reduction: *EVALCCD* (Duisenberg *et al.*, 2003 ▶); program(s) used to solve structure: *SHELXS97* (Sheldrick, 2008 ▶); program(s) used to refine structure: *SHELXL97* (Sheldrick, 2008 ▶); molecular graphics: *ORTEP-3 for Windows* (Farrugia, 1997 ▶) and *Mercury* (Macrae *et al.*, 2006 ▶); software used to prepare material for publication: *WinGX* publication routines (Farrugia, 1999 ▶).

## Supplementary Material

Crystal structure: contains datablock(s) global, I. DOI: 10.1107/S1600536811038682/bh2363sup1.cif

Structure factors: contains datablock(s) I. DOI: 10.1107/S1600536811038682/bh2363Isup2.hkl

Additional supplementary materials:  crystallographic information; 3D view; checkCIF report

## Figures and Tables

**Table:** 

Zn—O2*A*	1.981 (2)
Zn—O2*B*	2.017 (2)
Zn—O1*W*	2.034 (2)
Zn—N1*C*	2.098 (3)
Zn—O1*A*	2.275 (2)
Zn—O1*B*	2.283 (2)

**Table:** 

O2*A*—Zn—O2*B*	163.59 (9)
O2*A*—Zn—O1*W*	88.76 (10)
O2*B*—Zn—O1*W*	97.85 (9)
O2*A*—Zn—N1*C*	101.78 (10)
O2*B*—Zn—N1*C*	92.45 (10)
O1*W*—Zn—N1*C*	96.25 (10)
O2*A*—Zn—O1*A*	76.55 (9)
O2*B*—Zn—O1*A*	95.40 (9)
O1*W*—Zn—O1*A*	164.84 (8)
O2*B*—Zn—O1*B*	75.22 (8)
N1*C*—Zn—O1*B*	165.07 (9)

**Table 2:** Hydrogen-bond geometry (Å, °)

*D*—H⋯*A*	*D*—H	H⋯*A*	*D*⋯*A*	*D*—H⋯*A*
O1*W*—H11*W*⋯N1*D*^i^	0.85	1.97	2.754 (4)	153
O1*W*—H21*W*⋯O2*B*^ii^	0.86	1.97	2.750 (3)	150
O3*W*—H23*W*⋯N2*D*^iii^	0.83	2.06	2.887 (4)	173
O2*W*—H22*W*⋯N2*C*^iv^	0.83	2.10	2.863 (4)	152
O3*W*—H13*W*⋯O3*B*^ii^	0.83	2.02	2.846 (4)	175

Symmetry codes: (i) 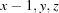; (ii) 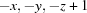; (iii) 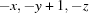; (iv) 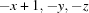.

**Fig. 1.**
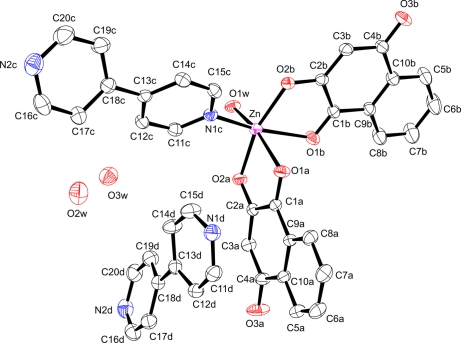
The molecular structure of the title compound, with 30% probability displacement ellipsoids.

**Fig. 2.**
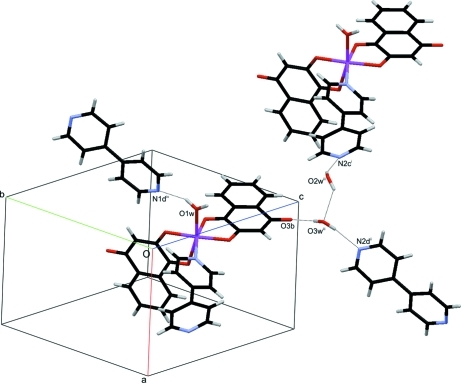
Hydrogen-bonds observed in the crystal packing. Symmetry codes: (i) -1+x, y, 1+z; (ii) x-1, y, 1+z; (iii) -x, -y, 1-z; (iv) -1+x, y, z.

## supplementary crystallographic information

### Comment

Quinones are a wide class of compounds with electroactivity, which coordinate to transition metal ions and can undergo metal-to-ligand electron transfer. The electrochemical properties of natural and synthetic naphthoquinones have been explored for applications such as the development of new drugs for cancer (Francisco *et al.*, 2008; Bustamante *et al.*, 2009; Neves *et al.*, 2009). Catechols and naphthoquinones have also been applied in studies related to molecular electronics (Beni *et al.*, 2006; Garge *et al.*, 1990). Impelled by these motivations, our group has started a systematic investigation on the coordination chemistry of 2-hydroxy-1,4-naphthoquinones.

The title compound (Figure 1) contains two lawsonate ligands coordinated in *cis* fashion by the O1A, O2A, O1B and O2B atoms with Zn—O bond lengths of 2.275 (5), 1.981 (2), 2.283 (2), and 2.017 (2) Å, respectively (table 1). One 4,4'-bipyridine and one water molecule complete the coordination sphere of the metal center. The *trans* angles of the metal center [N1C—Zn—O1B = 165.07 (9)°, O2B—Zn—O2A = 163.59 (9)° and O1A—Zn—O1W = 164.84 (8)°] indicate a deviation from the octahedral geometry. The asymmetric unit contains also one free 4,4'-bipyridine and two water molecules uncoordinated, which play a key role in the crystal packing. A 2D supramolecular network formed by O—H···N and O—H···O hydrogen bonds ([-1 0 1] and [-1 1 -1] directions) is observed (Figure 2, table 2).

### Experimental

All the reactants and solvents were obtained commercially, and used without any previous treatment. A methanolic solution (15 ml) containing lawsone (2 mmol, 0.3492 g) and triethylamine (2 mmol, 0.27 ml) was poured to a methanolic solution (10 ml) of zinc acetate (1 mmol, 0.2195 g) under magnetic stirring. To the reaction mixture, 4,4'-bipyridine (3 mmol, 0.4686 g) previously dissolved in 5 ml of MeOH was added dropwise. After 3 h at room temperature, a precipitate was formed and removed by filtration. The main solution was left undisturbed for a few days to produce red single crystals.

### Refinement

The positions of H atoms of the water molecules were found in difference maps and their positions fixed. Other H atoms were placed in calculated idealized positions, and refined as riding to their carrier atoms, with C—H bond lengths fixed to 0.93 Å. All H atoms were refined with fixed individual displacement parameters: *U*_iso_(H) = 1.2 *U*_eq_(carrier C) or 1.5 *U*_eq_ (carrier O).

### Crystal data

**Table:**

[Zn(C_10_H_5_O_3_)_2_(C_10_H_8_N_2_)(H_2_O)]·C_10_H_8_N_2_·2H_2_O	*V* = 1764.8 (6) Å^3^
*M**_r_* = 778.07	*Z* = 2
Triclinic, *P*1	*F*(000) = 804
Hall symbol: -P 1	*D*_x_ = 1.464 Mg m^−^^3^
*a* = 8.1448 (16) Å	Mo *K*α radiation, λ = 0.71073 Å
*b* = 14.179 (3) Å	θ = 3–25.0°
*c* = 15.910 (3) Å	µ = 0.76 mm^−^^1^
α = 73.90 (3)°	*T* = 293 K
β = 88.59 (3)°	Prism, red
γ = 89.53 (3)°	0.18 × 0.12 × 0.10 mm

### Data collection

**Table:**

Nonius Kappa CCD diffractometer	6230 independent reflections
Radiation source: Enraf Nonius FR590	3619 reflections with *I* > 2σ(*I*)
graphite	*R*_int_ = 0.060
Detector resolution: 9 pixels mm^-1^	θ_max_ = 25.0°, θ_min_ = 3°
ω scans	*h* = −9→9
Absorption correction: multi-scan (*SORTAV*; Blessing, 1995)	*k* = −16→16
*T*_min_ = 0.983, *T*_max_ = 1.000	*l* = −18→18
11283 measured reflections	

### Refinement

**Table:**

Refinement on *F*^2^	Primary atom site location: structure-invariant direct methods
Least-squares matrix: full	Secondary atom site location: difference Fourier map
*R*[*F*^2^ > 2σ(*F*^2^)] = 0.044	Hydrogen site location: inferred from neighbouring sites
*wR*(*F*^2^) = 0.089	H-atom parameters constrained
*S* = 0.91	*w* = 1/[σ^2^(*F*_o_^2^) + (0.0191*P*)^2^] where *P* = (*F*_o_^2^ + 2*F*_c_^2^)/3
6230 reflections	(Δ/σ)_max_ < 0.001
487 parameters	Δρ_max_ = 0.26 e Å^−^^3^
0 restraints	Δρ_min_ = −0.42 e Å^−^^3^
0 constraints	

### Fractional atomic coordinates and isotropic or equivalent isotropic displacement parameters (Å^2^)

**Table:**

	*x*	*y*	*z*	*U*_iso_*/*U*_eq_	
Zn	0.14452 (5)	0.16078 (3)	0.46808 (3)	0.03812 (13)	
O1A	0.3920 (3)	0.22621 (16)	0.48400 (14)	0.0428 (6)	
O1B	0.0717 (3)	0.22033 (16)	0.58312 (15)	0.0524 (7)	
O2B	0.1622 (3)	0.04451 (14)	0.57458 (14)	0.0392 (6)	
O3B	0.0658 (3)	−0.07129 (18)	0.87628 (16)	0.0629 (8)	
O1W	−0.0919 (3)	0.14001 (15)	0.43888 (14)	0.0427 (6)	
H11W	−0.1601	0.1805	0.4087	0.064*	
H21W	−0.1384	0.0833	0.4501	0.064*	
O3A	0.3758 (3)	0.60568 (17)	0.31208 (19)	0.0729 (9)	
O2A	0.1332 (3)	0.29577 (15)	0.38886 (14)	0.0402 (6)	
N1C	0.2534 (3)	0.08372 (19)	0.38604 (17)	0.0371 (7)	
N2C	0.6194 (4)	−0.1645 (3)	0.1018 (2)	0.0692 (10)	
C1B	0.0663 (4)	0.1554 (2)	0.6521 (2)	0.0374 (8)	
C1A	0.3950 (4)	0.3134 (2)	0.4424 (2)	0.0326 (8)	
C2B	0.1194 (4)	0.0545 (2)	0.6502 (2)	0.0348 (8)	
C2A	0.2478 (4)	0.3562 (2)	0.39009 (19)	0.0317 (8)	
C3B	0.1187 (4)	−0.0179 (2)	0.7269 (2)	0.0406 (9)	
H3B	0.1535	−0.0802	0.7258	0.049*	
C3A	0.2465 (4)	0.4537 (2)	0.3494 (2)	0.0398 (9)	
H3A	0.1529	0.4803	0.3194	0.048*	
C4B	0.0681 (4)	−0.0039 (3)	0.8082 (2)	0.0427 (9)	
C4A	0.3798 (5)	0.5179 (2)	0.3498 (2)	0.0441 (9)	
C5B	−0.0437 (5)	0.1123 (3)	0.8874 (2)	0.0604 (11)	
H5B	−0.0465	0.0607	0.9384	0.073*	
C5A	0.6636 (5)	0.5338 (3)	0.3969 (2)	0.0486 (10)	
H5A	0.6589	0.6005	0.3684	0.058*	
C6B	−0.0939 (6)	0.2042 (3)	0.8899 (3)	0.0767 (15)	
H6B	−0.1283	0.2149	0.9427	0.092*	
C6A	0.8045 (5)	0.4939 (3)	0.4380 (3)	0.0547 (10)	
H6A	0.8954	0.5336	0.4364	0.066*	
C7B	−0.0933 (6)	0.2801 (3)	0.8148 (3)	0.0837 (16)	
H7B	−0.1284	0.3421	0.8166	0.1*	
C7A	0.8122 (5)	0.3962 (3)	0.4812 (2)	0.0533 (10)	
H7A	0.9082	0.3701	0.5089	0.064*	
C8B	−0.0413 (5)	0.2652 (3)	0.7372 (2)	0.0634 (12)	
H8B	−0.0418	0.3169	0.6863	0.076*	
C8A	0.6787 (4)	0.3363 (3)	0.4839 (2)	0.0435 (9)	
H8A	0.684	0.2699	0.5135	0.052*	
C9B	0.0123 (4)	0.1728 (2)	0.7340 (2)	0.0403 (9)	
C9A	0.5369 (4)	0.3756 (2)	0.4421 (2)	0.0322 (8)	
C10B	0.0103 (4)	0.0959 (2)	0.8108 (2)	0.0427 (9)	
C10A	0.5293 (4)	0.4749 (2)	0.3980 (2)	0.0369 (8)	
C11C	0.2349 (4)	0.1148 (2)	0.2998 (2)	0.0419 (9)	
H11C	0.1715	0.1706	0.2774	0.05*	
C12C	0.3042 (4)	0.0690 (2)	0.2422 (2)	0.0418 (9)	
H12C	0.2879	0.094	0.1825	0.05*	
C13C	0.3984 (4)	−0.0143 (2)	0.2733 (2)	0.0363 (8)	
C14C	0.4180 (4)	−0.0468 (2)	0.3625 (2)	0.0414 (9)	
H14C	0.48	−0.1027	0.3866	0.05*	
C15C	0.3453 (4)	0.0038 (2)	0.4153 (2)	0.0413 (9)	
H15C	0.3611	−0.019	0.4752	0.05*	
C16C	0.6154 (5)	−0.0671 (3)	0.0819 (3)	0.0632 (12)	
H16C	0.6645	−0.0318	0.0293	0.076*	
C17C	0.5427 (5)	−0.0152 (3)	0.1345 (2)	0.0519 (10)	
H17C	0.5392	0.0531	0.1165	0.062*	
C18C	0.4754 (4)	−0.0665 (3)	0.2144 (2)	0.0429 (9)	
C19C	0.4820 (5)	−0.1670 (3)	0.2350 (3)	0.0605 (11)	
H19C	0.4378	−0.2045	0.2881	0.073*	
C20C	0.5531 (6)	−0.2123 (3)	0.1780 (3)	0.0744 (13)	
H20C	0.5549	−0.2805	0.1937	0.089*	
N1D	0.7606 (4)	0.2677 (2)	0.29807 (19)	0.0489 (8)	
N2D	0.2179 (5)	0.5997 (3)	0.0219 (2)	0.0646 (10)	
C11D	0.7972 (5)	0.3623 (3)	0.2675 (2)	0.0503 (10)	
H11D	0.8964	0.3836	0.2834	0.06*	
C12D	0.6980 (5)	0.4307 (3)	0.2138 (2)	0.0471 (9)	
H12D	0.7298	0.4962	0.1953	0.056*	
C13D	0.5517 (4)	0.4021 (2)	0.1872 (2)	0.0404 (9)	
C14D	0.5162 (5)	0.3032 (3)	0.2167 (2)	0.0531 (10)	
H14D	0.4208	0.2789	0.1995	0.064*	
C15D	0.6214 (5)	0.2416 (3)	0.2708 (3)	0.0561 (10)	
H15D	0.5926	0.1756	0.2902	0.067*	
C16D	0.3735 (6)	0.6222 (3)	0.0260 (2)	0.0621 (12)	
H16D	0.4097	0.6828	−0.0088	0.074*	
C17D	0.4865 (5)	0.5621 (3)	0.0784 (2)	0.0518 (10)	
H17D	0.5951	0.582	0.0778	0.062*	
C18D	0.4373 (5)	0.4722 (2)	0.1317 (2)	0.0421 (9)	
C19D	0.2736 (5)	0.4492 (3)	0.1290 (2)	0.0543 (10)	
H19D	0.2332	0.3899	0.1643	0.065*	
C20D	0.1709 (5)	0.5133 (3)	0.0747 (3)	0.0620 (11)	
H20D	0.0612	0.4956	0.0745	0.074*	
O2W	0.3076 (4)	0.2352 (2)	0.04695 (19)	0.0889 (10)	
H12W	0.2224	0.2686	0.0364	0.133*	
H22W	0.3141	0.1971	0.0156	0.133*	
O3W	−0.0082 (4)	0.27380 (19)	0.10522 (18)	0.0754 (9)	
H13W	−0.0245	0.2155	0.1071	0.113*	
H23W	−0.062	0.3104	0.0654	0.113*	

### Atomic displacement parameters (Å^2^)

**Table:**

	*U*^11^	*U*^22^	*U*^33^	*U*^12^	*U*^13^	*U*^23^
Zn	0.0433 (3)	0.0308 (2)	0.0384 (2)	−0.00580 (18)	−0.00147 (19)	−0.00647 (18)
O1A	0.0413 (15)	0.0313 (13)	0.0491 (15)	−0.0053 (11)	−0.0109 (11)	0.0012 (11)
O1B	0.080 (2)	0.0336 (14)	0.0383 (15)	0.0074 (13)	0.0006 (13)	−0.0022 (12)
O2B	0.0483 (15)	0.0304 (13)	0.0379 (14)	−0.0036 (11)	0.0020 (11)	−0.0081 (11)
O3B	0.091 (2)	0.0451 (16)	0.0410 (15)	−0.0023 (15)	0.0005 (14)	0.0076 (13)
O1W	0.0348 (14)	0.0346 (13)	0.0569 (15)	−0.0066 (11)	−0.0068 (11)	−0.0090 (11)
O3A	0.065 (2)	0.0290 (15)	0.109 (2)	−0.0066 (14)	−0.0223 (17)	0.0074 (15)
O2A	0.0391 (15)	0.0342 (13)	0.0432 (14)	−0.0077 (11)	−0.0079 (11)	−0.0031 (11)
N1C	0.0349 (17)	0.0341 (16)	0.0407 (18)	−0.0049 (13)	−0.0024 (14)	−0.0075 (14)
N2C	0.067 (3)	0.078 (3)	0.071 (3)	0.005 (2)	0.006 (2)	−0.035 (2)
C1B	0.038 (2)	0.040 (2)	0.033 (2)	0.0025 (17)	−0.0055 (16)	−0.0066 (17)
C1A	0.037 (2)	0.0290 (19)	0.0322 (18)	0.0000 (16)	−0.0002 (15)	−0.0096 (15)
C2B	0.031 (2)	0.0321 (19)	0.040 (2)	−0.0036 (15)	−0.0031 (16)	−0.0068 (17)
C2A	0.032 (2)	0.0325 (19)	0.0307 (19)	0.0017 (16)	−0.0005 (15)	−0.0088 (15)
C3B	0.046 (2)	0.0296 (19)	0.044 (2)	0.0029 (16)	−0.0074 (17)	−0.0061 (17)
C3A	0.034 (2)	0.036 (2)	0.048 (2)	0.0066 (17)	−0.0099 (17)	−0.0077 (17)
C4B	0.046 (2)	0.041 (2)	0.037 (2)	−0.0053 (18)	−0.0025 (18)	−0.0040 (18)
C4A	0.049 (2)	0.030 (2)	0.050 (2)	−0.0027 (18)	−0.0041 (19)	−0.0050 (17)
C5B	0.074 (3)	0.067 (3)	0.035 (2)	0.018 (2)	−0.005 (2)	−0.005 (2)
C5A	0.057 (3)	0.035 (2)	0.055 (2)	−0.0107 (19)	−0.002 (2)	−0.0137 (18)
C6B	0.102 (4)	0.089 (3)	0.042 (3)	0.042 (3)	−0.002 (2)	−0.025 (3)
C6A	0.041 (2)	0.060 (3)	0.068 (3)	−0.011 (2)	−0.013 (2)	−0.024 (2)
C7B	0.133 (5)	0.067 (3)	0.056 (3)	0.044 (3)	−0.010 (3)	−0.024 (3)
C7A	0.038 (2)	0.064 (3)	0.062 (3)	−0.002 (2)	−0.014 (2)	−0.022 (2)
C8B	0.097 (3)	0.051 (3)	0.042 (2)	0.025 (2)	−0.005 (2)	−0.014 (2)
C8A	0.041 (2)	0.042 (2)	0.050 (2)	0.0005 (18)	−0.0079 (18)	−0.0144 (18)
C9B	0.047 (2)	0.038 (2)	0.035 (2)	0.0049 (17)	−0.0040 (17)	−0.0086 (16)
C9A	0.031 (2)	0.0296 (18)	0.0362 (19)	−0.0034 (15)	0.0009 (15)	−0.0092 (15)
C10B	0.042 (2)	0.046 (2)	0.038 (2)	0.0049 (18)	−0.0042 (17)	−0.0075 (18)
C10A	0.038 (2)	0.0345 (19)	0.040 (2)	−0.0068 (17)	0.0032 (17)	−0.0147 (16)
C11C	0.042 (2)	0.036 (2)	0.045 (2)	0.0004 (17)	−0.0039 (18)	−0.0052 (18)
C12C	0.047 (2)	0.042 (2)	0.034 (2)	−0.0040 (18)	−0.0035 (17)	−0.0055 (17)
C13C	0.034 (2)	0.0338 (19)	0.040 (2)	−0.0080 (16)	0.0000 (16)	−0.0093 (16)
C14C	0.039 (2)	0.039 (2)	0.043 (2)	0.0036 (17)	0.0017 (17)	−0.0064 (18)
C15C	0.037 (2)	0.049 (2)	0.035 (2)	−0.0024 (18)	−0.0038 (17)	−0.0040 (17)
C16C	0.053 (3)	0.087 (3)	0.046 (2)	−0.002 (2)	0.009 (2)	−0.013 (2)
C17C	0.055 (3)	0.057 (2)	0.044 (2)	0.001 (2)	0.002 (2)	−0.014 (2)
C18C	0.037 (2)	0.046 (2)	0.046 (2)	−0.0044 (17)	−0.0052 (18)	−0.0137 (19)
C19C	0.071 (3)	0.046 (2)	0.065 (3)	−0.005 (2)	0.014 (2)	−0.017 (2)
C20C	0.082 (3)	0.060 (3)	0.088 (4)	−0.002 (2)	0.017 (3)	−0.033 (3)
N1D	0.047 (2)	0.049 (2)	0.0489 (19)	−0.0013 (16)	−0.0065 (16)	−0.0111 (16)
N2D	0.076 (3)	0.063 (2)	0.055 (2)	0.021 (2)	−0.013 (2)	−0.0169 (19)
C11D	0.045 (2)	0.053 (3)	0.053 (2)	−0.007 (2)	−0.0088 (19)	−0.016 (2)
C12D	0.055 (3)	0.039 (2)	0.045 (2)	−0.0064 (19)	−0.0050 (19)	−0.0068 (18)
C13D	0.045 (2)	0.041 (2)	0.036 (2)	0.0044 (18)	−0.0026 (17)	−0.0123 (17)
C14D	0.043 (2)	0.040 (2)	0.072 (3)	−0.0028 (19)	−0.015 (2)	−0.008 (2)
C15D	0.050 (3)	0.038 (2)	0.075 (3)	−0.003 (2)	−0.011 (2)	−0.007 (2)
C16D	0.089 (4)	0.043 (2)	0.050 (3)	0.013 (3)	0.004 (3)	−0.008 (2)
C17D	0.059 (3)	0.044 (2)	0.053 (2)	0.003 (2)	−0.002 (2)	−0.014 (2)
C18D	0.048 (3)	0.041 (2)	0.037 (2)	−0.0020 (19)	−0.0007 (18)	−0.0122 (17)
C19D	0.057 (3)	0.050 (2)	0.053 (2)	0.003 (2)	−0.011 (2)	−0.008 (2)
C20D	0.062 (3)	0.067 (3)	0.058 (3)	0.006 (2)	−0.016 (2)	−0.016 (2)
O2W	0.087 (2)	0.108 (3)	0.080 (2)	−0.0004 (19)	−0.0010 (18)	−0.039 (2)
O3W	0.083 (2)	0.0636 (19)	0.080 (2)	0.0080 (16)	−0.0140 (17)	−0.0199 (16)

### Geometric parameters (Å, °)

**Table:**

Zn—O2A	1.981 (2)	C8A—C9A	1.381 (4)
Zn—O2B	2.017 (2)	C8A—H8A	0.93
Zn—O1W	2.034 (2)	C9B—C10B	1.394 (4)
Zn—N1C	2.098 (3)	C9A—C10A	1.391 (4)
Zn—O1A	2.275 (2)	C11C—C12C	1.370 (4)
Zn—O1B	2.283 (2)	C11C—H11C	0.93
O1A—C1A	1.230 (3)	C12C—C13C	1.382 (4)
O1B—C1B	1.221 (4)	C12C—H12C	0.93
O2B—C2B	1.290 (4)	C13C—C14C	1.378 (4)
O3B—C4B	1.230 (4)	C13C—C18C	1.473 (4)
O1W—H11W	0.8505	C14C—C15C	1.369 (4)
O1W—H21W	0.8632	C14C—H14C	0.93
O3A—C4A	1.223 (4)	C15C—H15C	0.93
O2A—C2A	1.277 (4)	C16C—C17C	1.380 (5)
N1C—C15C	1.331 (4)	C16C—H16C	0.93
N1C—C11C	1.332 (4)	C17C—C18C	1.380 (5)
N2C—C20C	1.319 (5)	C17C—H17C	0.93
N2C—C16C	1.328 (5)	C18C—C19C	1.372 (5)
C1B—C9B	1.450 (4)	C19C—C20C	1.364 (5)
C1B—C2B	1.499 (4)	C19C—H19C	0.93
C1A—C9A	1.458 (4)	C20C—H20C	0.93
C1A—C2A	1.502 (4)	N1D—C15D	1.316 (4)
C2B—C3B	1.359 (4)	N1D—C11D	1.327 (4)
C2A—C3A	1.354 (4)	N2D—C16D	1.317 (5)
C3B—C4B	1.413 (4)	N2D—C20D	1.332 (5)
C3B—H3B	0.93	C11D—C12D	1.373 (4)
C3A—C4A	1.424 (5)	C11D—H11D	0.93
C3A—H3A	0.93	C12D—C13D	1.375 (5)
C4B—C10B	1.499 (5)	C12D—H12D	0.93
C4A—C10A	1.489 (4)	C13D—C14D	1.380 (5)
C5B—C10B	1.364 (5)	C13D—C18D	1.475 (5)
C5B—C6B	1.373 (5)	C14D—C15D	1.358 (5)
C5B—H5B	0.93	C14D—H14D	0.93
C5A—C6A	1.373 (5)	C15D—H15D	0.93
C5A—C10A	1.378 (5)	C16D—C17D	1.378 (5)
C5A—H5A	0.93	C16D—H16D	0.93
C6B—C7B	1.368 (5)	C17D—C18D	1.377 (5)
C6B—H6B	0.93	C17D—H17D	0.93
C6A—C7A	1.366 (5)	C18D—C19D	1.380 (5)
C6A—H6A	0.93	C19D—C20D	1.364 (5)
C7B—C8B	1.366 (5)	C19D—H19D	0.93
C7B—H7B	0.93	C20D—H20D	0.93
C7A—C8A	1.379 (5)	O2W—H12W	0.83
C7A—H7A	0.93	O2W—H22W	0.83
C8B—C9B	1.390 (4)	O3W—H13W	0.83
C8B—H8B	0.93	O3W—H23W	0.83
			
O2A—Zn—O2B	163.59 (9)	C8B—C9B—C1B	121.0 (3)
O2A—Zn—O1W	88.76 (10)	C10B—C9B—C1B	120.0 (3)
O2B—Zn—O1W	97.85 (9)	C8A—C9A—C10A	120.0 (3)
O2A—Zn—N1C	101.78 (10)	C8A—C9A—C1A	120.6 (3)
O2B—Zn—N1C	92.45 (10)	C10A—C9A—C1A	119.4 (3)
O1W—Zn—N1C	96.25 (10)	C5B—C10B—C9B	119.7 (3)
O2A—Zn—O1A	76.55 (9)	C5B—C10B—C4B	120.8 (3)
O2B—Zn—O1A	95.40 (9)	C9B—C10B—C4B	119.5 (3)
O1W—Zn—O1A	164.84 (8)	C5A—C10A—C9A	119.5 (3)
N1C—Zn—O1A	90.48 (9)	C5A—C10A—C4A	119.6 (3)
O2A—Zn—O1B	89.43 (9)	C9A—C10A—C4A	120.8 (3)
O2B—Zn—O1B	75.22 (8)	N1C—C11C—C12C	123.5 (3)
O1W—Zn—O1B	93.79 (9)	N1C—C11C—H11C	118.2
N1C—Zn—O1B	165.07 (9)	C12C—C11C—H11C	118.2
O1A—Zn—O1B	82.50 (9)	C11C—C12C—C13C	119.7 (3)
C1A—O1A—Zn	109.5 (2)	C11C—C12C—H12C	120.2
C1B—O1B—Zn	111.8 (2)	C13C—C12C—H12C	120.2
C2B—O2B—Zn	119.13 (18)	C14C—C13C—C12C	117.0 (3)
Zn—O1W—H11W	129.9	C14C—C13C—C18C	121.0 (3)
Zn—O1W—H21W	124	C12C—C13C—C18C	121.9 (3)
H11W—O1W—H21W	105.6	C15C—C14C—C13C	119.4 (3)
C2A—O2A—Zn	119.0 (2)	C15C—C14C—H14C	120.3
C15C—N1C—C11C	116.3 (3)	C13C—C14C—H14C	120.3
C15C—N1C—Zn	123.3 (2)	N1C—C15C—C14C	124.0 (3)
C11C—N1C—Zn	120.3 (2)	N1C—C15C—H15C	118
C20C—N2C—C16C	116.4 (4)	C14C—C15C—H15C	118
O1B—C1B—C9B	122.6 (3)	N2C—C16C—C17C	124.0 (4)
O1B—C1B—C2B	117.5 (3)	N2C—C16C—H16C	118
C9B—C1B—C2B	119.8 (3)	C17C—C16C—H16C	118
O1A—C1A—C9A	122.0 (3)	C18C—C17C—C16C	118.6 (4)
O1A—C1A—C2A	118.5 (3)	C18C—C17C—H17C	120.7
C9A—C1A—C2A	119.4 (3)	C16C—C17C—H17C	120.7
O2B—C2B—C3B	125.7 (3)	C19C—C18C—C17C	117.0 (3)
O2B—C2B—C1B	116.1 (3)	C19C—C18C—C13C	122.3 (3)
C3B—C2B—C1B	118.2 (3)	C17C—C18C—C13C	120.7 (3)
O2A—C2A—C3A	125.8 (3)	C20C—C19C—C18C	120.3 (4)
O2A—C2A—C1A	115.8 (3)	C20C—C19C—H19C	119.9
C3A—C2A—C1A	118.4 (3)	C18C—C19C—H19C	119.9
C2B—C3B—C4B	123.7 (3)	N2C—C20C—C19C	123.6 (4)
C2B—C3B—H3B	118.1	N2C—C20C—H20C	118.2
C4B—C3B—H3B	118.1	C19C—C20C—H20C	118.2
C2A—C3A—C4A	124.0 (3)	C15D—N1D—C11D	115.0 (3)
C2A—C3A—H3A	118	C16D—N2D—C20D	115.6 (4)
C4A—C3A—H3A	118	N1D—C11D—C12D	124.2 (4)
O3B—C4B—C3B	122.3 (3)	N1D—C11D—H11D	117.9
O3B—C4B—C10B	118.9 (3)	C12D—C11D—H11D	117.9
C3B—C4B—C10B	118.8 (3)	C11D—C12D—C13D	119.9 (3)
O3A—C4A—C3A	122.7 (3)	C11D—C12D—H12D	120.1
O3A—C4A—C10A	119.5 (3)	C13D—C12D—H12D	120.1
C3A—C4A—C10A	117.8 (3)	C12D—C13D—C14D	115.9 (3)
C10B—C5B—C6B	120.7 (4)	C12D—C13D—C18D	122.7 (3)
C10B—C5B—H5B	119.7	C14D—C13D—C18D	121.4 (3)
C6B—C5B—H5B	119.7	C15D—C14D—C13D	119.7 (4)
C6A—C5A—C10A	120.0 (3)	C15D—C14D—H14D	120.2
C6A—C5A—H5A	120	C13D—C14D—H14D	120.2
C10A—C5A—H5A	120	N1D—C15D—C14D	125.3 (4)
C7B—C6B—C5B	120.0 (4)	N1D—C15D—H15D	117.4
C7B—C6B—H6B	120	C14D—C15D—H15D	117.4
C5B—C6B—H6B	120	N2D—C16D—C17D	124.5 (4)
C7A—C6A—C5A	120.6 (4)	N2D—C16D—H16D	117.8
C7A—C6A—H6A	119.7	C17D—C16D—H16D	117.8
C5A—C6A—H6A	119.7	C18D—C17D—C16D	119.5 (4)
C8B—C7B—C6B	120.3 (4)	C18D—C17D—H17D	120.3
C8B—C7B—H7B	119.8	C16D—C17D—H17D	120.3
C6B—C7B—H7B	119.8	C17D—C18D—C19D	116.2 (3)
C6A—C7A—C8A	120.4 (3)	C17D—C18D—C13D	122.8 (4)
C6A—C7A—H7A	119.8	C19D—C18D—C13D	120.9 (3)
C8A—C7A—H7A	119.8	C20D—C19D—C18D	120.1 (4)
C7B—C8B—C9B	120.2 (4)	C20D—C19D—H19D	119.9
C7B—C8B—H8B	119.9	C18D—C19D—H19D	119.9
C9B—C8B—H8B	119.9	N2D—C20D—C19D	124.0 (4)
C7A—C8A—C9A	119.5 (3)	N2D—C20D—H20D	118
C7A—C8A—H8A	120.3	C19D—C20D—H20D	118
C9A—C8A—H8A	120.3	H12W—O2W—H22W	110.0
C8B—C9B—C10B	119.0 (3)	H13W—O3W—H23W	110.0
			
O2A—Zn—O1A—C1A	5.02 (19)	C7A—C8A—C9A—C1A	178.7 (3)
O2B—Zn—O1A—C1A	−160.5 (2)	O1A—C1A—C9A—C8A	4.8 (5)
O1W—Zn—O1A—C1A	−9.6 (4)	C2A—C1A—C9A—C8A	−174.8 (3)
N1C—Zn—O1A—C1A	107.0 (2)	O1A—C1A—C9A—C10A	−176.3 (3)
O1B—Zn—O1A—C1A	−86.2 (2)	C2A—C1A—C9A—C10A	4.2 (4)
O2A—Zn—O1B—C1B	−178.7 (2)	C6B—C5B—C10B—C9B	−0.9 (6)
O2B—Zn—O1B—C1B	−4.6 (2)	C6B—C5B—C10B—C4B	178.9 (4)
O1W—Zn—O1B—C1B	92.5 (2)	C8B—C9B—C10B—C5B	−0.1 (5)
N1C—Zn—O1B—C1B	−39.7 (5)	C1B—C9B—C10B—C5B	−178.7 (3)
O1A—Zn—O1B—C1B	−102.2 (2)	C8B—C9B—C10B—C4B	−179.9 (3)
O2A—Zn—O2B—C2B	24.9 (5)	C1B—C9B—C10B—C4B	1.5 (5)
O1W—Zn—O2B—C2B	−88.2 (2)	O3B—C4B—C10B—C5B	0.2 (5)
N1C—Zn—O2B—C2B	175.2 (2)	C3B—C4B—C10B—C5B	178.3 (3)
O1A—Zn—O2B—C2B	84.5 (2)	O3B—C4B—C10B—C9B	−180.0 (3)
O1B—Zn—O2B—C2B	3.7 (2)	C3B—C4B—C10B—C9B	−1.9 (5)
O2B—Zn—O2A—C2A	54.2 (4)	C6A—C5A—C10A—C9A	1.0 (5)
O1W—Zn—O2A—C2A	168.4 (2)	C6A—C5A—C10A—C4A	−177.2 (3)
N1C—Zn—O2A—C2A	−95.4 (2)	C8A—C9A—C10A—C5A	−0.5 (5)
O1A—Zn—O2A—C2A	−7.8 (2)	C1A—C9A—C10A—C5A	−179.4 (3)
O1B—Zn—O2A—C2A	74.6 (2)	C8A—C9A—C10A—C4A	177.7 (3)
O2A—Zn—N1C—C15C	151.6 (3)	C1A—C9A—C10A—C4A	−1.2 (4)
O2B—Zn—N1C—C15C	−20.2 (3)	O3A—C4A—C10A—C5A	−2.3 (5)
O1W—Zn—N1C—C15C	−118.4 (3)	C3A—C4A—C10A—C5A	177.3 (3)
O1A—Zn—N1C—C15C	75.2 (3)	O3A—C4A—C10A—C9A	179.5 (3)
O1B—Zn—N1C—C15C	13.6 (6)	C3A—C4A—C10A—C9A	−1.0 (5)
O2A—Zn—N1C—C11C	−27.3 (3)	C15C—N1C—C11C—C12C	0.1 (5)
O2B—Zn—N1C—C11C	161.0 (3)	Zn—N1C—C11C—C12C	179.1 (3)
O1W—Zn—N1C—C11C	62.8 (3)	N1C—C11C—C12C—C13C	0.3 (5)
O1A—Zn—N1C—C11C	−103.6 (3)	C11C—C12C—C13C—C14C	−0.3 (5)
O1B—Zn—N1C—C11C	−165.2 (3)	C11C—C12C—C13C—C18C	179.9 (3)
Zn—O1B—C1B—C9B	−175.7 (3)	C12C—C13C—C14C—C15C	−0.2 (5)
Zn—O1B—C1B—C2B	4.7 (4)	C18C—C13C—C14C—C15C	179.6 (3)
Zn—O1A—C1A—C9A	178.3 (2)	C11C—N1C—C15C—C14C	−0.7 (5)
Zn—O1A—C1A—C2A	−2.1 (3)	Zn—N1C—C15C—C14C	−179.6 (3)
Zn—O2B—C2B—C3B	177.4 (3)	C13C—C14C—C15C—N1C	0.7 (5)
Zn—O2B—C2B—C1B	−2.6 (4)	C20C—N2C—C16C—C17C	−2.0 (6)
O1B—C1B—C2B—O2B	−1.9 (5)	N2C—C16C—C17C—C18C	2.7 (6)
C9B—C1B—C2B—O2B	178.5 (3)	C16C—C17C—C18C—C19C	−1.6 (5)
O1B—C1B—C2B—C3B	178.1 (3)	C16C—C17C—C18C—C13C	179.2 (3)
C9B—C1B—C2B—C3B	−1.5 (5)	C14C—C13C—C18C—C19C	39.9 (5)
Zn—O2A—C2A—C3A	−170.3 (2)	C12C—C13C—C18C—C19C	−140.3 (4)
Zn—O2A—C2A—C1A	9.2 (3)	C14C—C13C—C18C—C17C	−141.0 (4)
O1A—C1A—C2A—O2A	−4.1 (4)	C12C—C13C—C18C—C17C	38.8 (5)
C9A—C1A—C2A—O2A	175.4 (3)	C17C—C18C—C19C—C20C	0.2 (6)
O1A—C1A—C2A—C3A	175.4 (3)	C13C—C18C—C19C—C20C	179.3 (4)
C9A—C1A—C2A—C3A	−5.0 (4)	C16C—N2C—C20C—C19C	0.4 (7)
O2B—C2B—C3B—C4B	−178.9 (3)	C18C—C19C—C20C—N2C	0.5 (7)
C1B—C2B—C3B—C4B	1.1 (5)	C15D—N1D—C11D—C12D	2.2 (5)
O2A—C2A—C3A—C4A	−177.6 (3)	N1D—C11D—C12D—C13D	−1.2 (5)
C1A—C2A—C3A—C4A	2.9 (5)	C11D—C12D—C13D—C14D	−1.0 (5)
C2B—C3B—C4B—O3B	178.6 (3)	C11D—C12D—C13D—C18D	178.2 (3)
C2B—C3B—C4B—C10B	0.5 (5)	C12D—C13D—C14D—C15D	2.1 (5)
C2A—C3A—C4A—O3A	179.5 (3)	C18D—C13D—C14D—C15D	−177.1 (3)
C2A—C3A—C4A—C10A	0.0 (5)	C11D—N1D—C15D—C14D	−1.0 (6)
C10B—C5B—C6B—C7B	1.3 (7)	C13D—C14D—C15D—N1D	−1.1 (6)
C10A—C5A—C6A—C7A	−0.9 (5)	C20D—N2D—C16D—C17D	1.8 (5)
C5B—C6B—C7B—C8B	−0.6 (8)	N2D—C16D—C17D—C18D	−0.8 (5)
C5A—C6A—C7A—C8A	0.2 (6)	C16D—C17D—C18D—C19D	−0.7 (5)
C6B—C7B—C8B—C9B	−0.4 (7)	C16D—C17D—C18D—C13D	177.7 (3)
C6A—C7A—C8A—C9A	0.3 (5)	C12D—C13D—C18D—C17D	22.7 (5)
C7B—C8B—C9B—C10B	0.8 (6)	C14D—C13D—C18D—C17D	−158.1 (3)
C7B—C8B—C9B—C1B	179.4 (4)	C12D—C13D—C18D—C19D	−159.0 (3)
O1B—C1B—C9B—C8B	2.0 (6)	C14D—C13D—C18D—C19D	20.2 (5)
C2B—C1B—C9B—C8B	−178.4 (3)	C17D—C18D—C19D—C20D	1.0 (5)
O1B—C1B—C9B—C10B	−179.4 (3)	C13D—C18D—C19D—C20D	−177.4 (3)
C2B—C1B—C9B—C10B	0.2 (5)	C16D—N2D—C20D—C19D	−1.4 (6)
C7A—C8A—C9A—C10A	−0.2 (5)	C18D—C19D—C20D—N2D	0.1 (6)

### Hydrogen-bond geometry (Å, °)

**Table:**

*D*—H···*A*	*D*—H	H···*A*	*D*···*A*	*D*—H···*A*
O2W—H12W···O3W	0.83	2.16	2.814 (4)	135
O1W—H11W···N1D^i^	0.85	1.97	2.754 (4)	153
O1W—H21W···O2B^ii^	0.86	1.97	2.750 (3)	150
O3W—H23W···N2D^iii^	0.83	2.06	2.887 (4)	173
O2W—H22W···N2C^iv^	0.83	2.10	2.863 (4)	152
O3W—H13W···O3B^ii^	0.83	2.02	2.846 (4)	175

Symmetry codes: (i) *x*−1, *y*, *z*; (ii) −*x*, −*y*, −*z*+1; (iii) −*x*, −*y*+1, −*z*; (iv) −*x*+1, −*y*, −*z*.
